# Hepatitis C virus screening and treatment in Irish prisons from nurse managers’ perspectives - a qualitative exploration

**DOI:** 10.1186/s12912-019-0347-x

**Published:** 2019-06-13

**Authors:** D. Crowley, M. C. Van Hout, C. Murphy, E. Kelly, J. S. Lambert, W. Cullen

**Affiliations:** 1 Irish College of General Practitioners, Lincoln Place, Dublin, Ireland; 2 Public Health Institute, Liverpool John Moore’s University, Liverpool, UK; 3 Irish Prison Service, Mountjoy Prison, Dublin 7, Ireland; 4 Department of Infectious Diseases, School of Medicine, University College Dublin, Mater Misericordiae University Hospital, Dublin, Ireland; 5 School of Medicine, University College Dublin, Dublin, Ireland

## Abstract

**Background:**

Prisoners carry a greater burden of physical, communicable and psychiatric disease compared to the general population. Prison health care structures are complex and provide challenges and opportunities to engage a marginalised and poorly served group with health care including Hepatitis C Virus (HCV) screening, assessment and treatment. Optimising HCV management in prisons is a public health priority. Nurses are the primary healthcare providers in most prisons globally. Understanding the barriers and facilitators to prisoners engaging in HCV care from the perspectives of nurses is the first step in implementing effective strategies to eliminate HCV from prison settings. The aim of this study was to identify the barriers and facilitators to HCV screening and treatment in Irish prisons from a nurse perspective and inform the implementation of a national prison-based HCV screening program.

**Methods:**

A qualitative study using focus group methodology underpinned by grounded theory for analysis in a national group of nurse managers (*n* = 12).

**Results:**

The following themes emerged from the analysis; security and safety requirements impacting patient access, staffing and rostering issues, prison nurses’ skill set and concerns around phlebotomy, conflict between maintaining confidentiality and concerns for personal safety, peer workers, prisoners’ lack of knowledge, fear of treatment and stigma, inter-prison variations in prisoner health needs and health service delivery and priority, linkage to care, timing of screening and stability of prison life.

**Conclusions:**

Prison nurses are uniquely placed to identify barriers and facilitators to HCV screening and treatment in prisoners and inform changes to health care practice and policy that will optimi**se** the public health opportunity that incarceration provides.

## Background

Hepatitis C virus (HCV) infection is a major public health problem, causing a significant amount of liver related morbidity and mortality globally [1, 2]. In Ireland, like other developed countries, injecting drug use (IDU) is now the main route of HCV transmission [3–6].

HCV infection of often described as a silent epidemic with less than half of those infected aware of their HCV status [7]. Recent advances in HCV treatment including, direct acting anti-virals (DAA), mobile elastography, less restrictive treatment guidelines and the movement of treatment out of hospital based specialist services have revolutionised the HCV treatment landscape [8–11]. The WHO have declared HCV elimination by 2030 a key public health target [12].

People who inject drugs (PWID) carry a much higher HCV disease burden than the general population with HCV prevalence estimates of over 50% [13–15]. The ongoing criminalisation of drug users ensures a high HCV prevalence among prison populations [16–19]. The prevention, identification and treatment of HCV infection have been identified as a key priority for prison healthcare [20, 21].

Prisoners have multiple risk factors for HCV acquisition including unsafe IDU, non-sterile tattooing, violent assault and the sharing of tooth brushes and hair clippers [16, 22, 23]. Sexual transmission between men who have sex with men (MSM) is also a concern in prison where condoms are not available [24]. A 2013 systematic review and meta-analysis reported a HCV incidence of 1.4 per 100-person years and 16.4 per 100-person years in detainees with a history of IDU [16] and a summary HCV prevalence estimate for general detainees of 26%, increasing to 64% in those with a history of IDU [16].

There are 3674 persons incarcerated in Ireland on any given day and the annual turnover of prisoners is 14,182 [25]. High levels of IDU have been reported in both male (42%) and female (60%) prisoners [26]. A 2000 study estimated a HCV prevalence of 37% in the Irish prison population increasing to 81% % in those with a history of IDU [27]. A later (2014) study found a reduced HCV prevalence of 13% [26]. HCV screening in Irish prisons is ad hoc and there is no data available on the numbers of prisoners screened or treated for HCV infection annually [28, 29]. Furthermore, there is no HCV- related educational material available to prisoners either at committal or during their period of incarceration [29, 30].

A number of studies (two Irish papers) have identified barriers and facilitators to HCV screening and treatment in PWID [31–35]. These barriers include; perceiving HCV infection to be a benign illness, having no symptoms, fear of investigations and treatment, lack of information on testing locations, not being referred for specialist care, ineligibility for treatment, competing priorities and fear of stigma. Enablers include; trusted relationships with health professionals, education on HCV infection and management, developing symptoms, family responsibilities, and wanting to progress from drug use. Prisoners experience many similar barriers and other unique ones related to incarceration, including a lack of proactive approaches to offering testing, fears and lack of knowledge about HCV infection, concerns about confidentiality and stigma, and lack of continuity of care in the event of transfer or release [29, 36, 37].

International and national guidelines on prisoner health recommend that HCV screening and treatment be made an integral part of all prison health care systems [21, 38]. Despite these recommendations and evidence that when made available HCV screening and treatment can be safely and successfully provided in prison settings, often prisoners are released back to their communities unaware of their HCV status and untreated [7].

Nurses provide the majority of health care in prisons and were introduced into the Irish prison health system in 1999 [39]. They currently provide medical care in all 15 Irish prisons. Each location has a designated nurse manager who provides clinical and operational governance to the nursing service. Nurses play a pivotal role in primary care delivery, addiction treatment and the management of blood borne virus (BBV) infections including HCV infection [39, 40].

This paper reports on the qualitative component of a larger European project to find and treat HCV infection among hard to reach vulnerable populations [41]. It complements the two previous Irish studies reporting barriers to HCV management and adds to the small number of international prison-based studies in this area [29, 30, 36, 42]. It is unique in reporting these from a nurse managers’ perspective and being national in coverage.

## Methodology

The nurse managers of all thirteen closed prisons in the Irish Prison Service (IPS) were invited to attend a focus group. This methodology was used because perspectives are more likely to be revealed by interaction and discussion with peers and for its utility in a real-life prison setting. Ethical approval was obtained from the Mater Ethics Committee, Dublin and supported and endorsed by the IPS Ethics group.

Following, a review of the literature on the topic, completion of a scoping review and consultation with the research group and national experts in the area a focus group guide was finalised. This guideline included a series of open-ended questions covering the following areas; experience of prison- based HCV screening and treatment, barriers and enablers to uptake, challenges related to incarceration and release, inter-prison variations in health care delivery and role of security staff and peers in prison HCV management.

Twelve of the thirteen nurse managers participated in the focus group. All participants were given a participant information leaflet (PIL) to read and had an opportunity to ask questions before giving their informed consent to participate. The focus group lasted for a period of 90 min. The group was moderated by researcher (DC) and observed by researcher (MCVH) from the research team. The focus group interview was recorded using an encrypted digital audio recorder, and observations of group dynamics and interactions were written in field notes during and after the event.

All audio recordings were transcribed using Microsoft word and were uploaded to NVIVO 11 for coding and thematic analysis. A grounded theory approach informed both the collection and analysis of the data. The thematic coding was revised with each analysis of the focus group narrative and analysis ceased when thematic saturation was achieved (agreed by researcher 1 and 2). Illustrative quotes from the recorded narratives supporting the thematic analysis are reported as either Dublin or Non-Dublin based. This is to protect the confidentiality of the participants.

## Results

The following themes emerged from analysis of the data; security and safety, staffing and rostering issues, skill set and concerns regarding phlebotomy skills, conflict between maintaining confidentiality and concerns for personal safety, peer networks, prisoners’ lack of knowledge, fear of treatment and stigma, inter-prison variations in prisoner health needs, health service delivery and priority, linkage to care, timing of screening and stability of prison life.

### Security and safety requirements impacting access to prisoners

Many participants described the limitations that security and safety within their prisons placed on health care delivery. Accessing prisoners was often problematic in particular protection prisoners with heightened security requirements. Participants unanimously agreed that their professional ethics were never compromised and reported that security staff respected their roles.

*“security comes first and we may never get to see the prisoners*”. (Dublin)

*“The access to patient group is so limited”.* (Non- Dublin)

*“We work well with the prison officers and governors, we respect each other’s roles ... I never feel compromised”.* (Dublin)

### Staffing and rostering issues

Nurse managers reported lack of staffing as a barrier to provision of health care in their prisons. The structured rostering system based a three-month cycle (a quarter) often created staff deficits towards the end of the cycle. A number of duties are prioritised during periods of short staffing which included the completion of the committal interview and the dispensing of medications. Blood borne virus screening was often “demoted” during these times.

*“We’re not flushed with staff to do it”.* (Non-Dublin).

*“With regard to screening, it’s not systematic it is very staff dependant”.* (Dublin).

*“Phlebotomy lists have definitely gone down on the priority list when we’re short staffed”*. (Non-Dublin).

*“We tend historically to be dangerously short of staff for protracted periods of time”.* **(**Non-Dublin).

*Generally, happens more at the start of each pool of overtime hours and rarely is the activity done at the end when you’re really short and you’re barely giving out the medication. So, it’s quite sporadic and opportunistic.* (Dublin).

### Skillset and concerns regarding phlebotomy

Focus group participants identified that nurses working in the IPS had a specific skill set that matched the complex health needs of prisoners and was suited to caring for HCV infected prisoners through the screening and treatment process. Knowledge of the local environment and how it impacting health care delivery was viewed as important.

*“There’s a particular skill set that is prison nursing ... knows the care plan of that individual, knows the dynamic of the environment and can support the patient through that treatment*”. (Dublin).

*“We have a wide variation of expertise amongst the nursing group .... many specialist interests as well. It makes perfect sense for infectious diseases or STIs; just as a cohort it makes perfect sense”.* (Non-Dublin).

Fear of performing phlebotomy was identified by a number of participants. This was linked with the known high levels of BBV infection among prisoners, and the associated risks of needle stick injury. and fear that prisoners might use the needle as a weapon. Many nurses had not received phlebotomy training and of those who did were seen to lack confidence in conducting the procedure.

*“There’s a problem of skill mix. Lots of nurses don’t take bloods. If you have nobody trained up that’s a problem. Some nurses are afraid to take bloods. There’s a fear. The virus itself ... people not confident taking bloods”.* (Dublin).

### Conflict between maintaining confidentiality and concerns for personal safety

Some participants expressed concerns about medical confidentiality in particular the presence of prison officers during the committal interview and during ward rounds. This was also linked with fear for personal safety. A number of participants expressed concerns about taking blood without the presence of security staff and the potential for needles to be used as weapons. Some participants felt conflicted between wishing to maintain confidentiality for the prisoners and the need to ensure personally safety.

*“I think confidentiality is a huge thing*”. (Dublin).

*“There are security issues though; I would not like to do it without an officer… I feel conflicted”.* (Non-Dublin).

*“Yeah it is a weapon too”.* (Non-Dublin).

*“There are some that you need two offices to deal with them so in that cases, different things for different prisoners”.* (Dublin).

### Peer networks

There were mixed views in the group about the use of peer workers in prison health care including HCV screening and treatment. Many participants had worked with peers in mass screening initiatives and were very positive about their benefits. Concerns were expressed with regard to confidentiality, the accuracy of information being provided, the structures required to ensure governance and maintenance of prison security.

*“We do a lot of screening through Red Cross initiatives* (Peer workers). *Suppose maybe we should do widespread screening as a once off time, might be worth it”.* (Dublin).

*“They are not that confidential with other prisoners*”. (Dublin).

*“I think there would be concerns … security is a real one… I* think *we need to be careful what we subcontract out. The information that is going to people … may not always be accurate. We have a responsibility when people are engaging with treatment that we’re providing really good information”*. (Dublin).

*“I know peer support can be great but we need to be careful with the role they can play and that we are not abdicating our role. The governance of it, the security issues, and the obligations we have must be considered you know”.* (Non-Dublin).

### Prisoners’ lack of knowledge, fear of treatment and stigma

Lack of knowledge among prisoners was seen as barriers to engagement with HCV care. This was also linked with fear of treatment including liver biopsy and interferon-based treatment regimes.

*“The myths are still out there and it goes to show maybe we need to follow up on that*”. (Dublin).

*“They (*prisoners) *can be the biggest block not because they are opposed to it but because they don’t have the knowledge”.* **(**Dublin).

*“Most of the prisoners don’t actually know the difference between hepatitis A, B, C or D. So if we ask have you hepatitis C? They say ‘what’s the difference”?* (Non-Dublin).

“Some of the stories about what the treatment is and how that affects people. I’ve heard people say they’ve relapsed because of the treatment, so there is that fear that comes into it, you know ‘is that treatment going to help or make me relapse”? (Dublin).

Prisoners’ concern around stigma was also seen as a block to prisoners engaging in HCV care. Many participants argued that making screening routine and universal had the potential to reduce this stigma. Participants were concerned about the maintenance of confidentiality and how this increased the fear that prisoners had about being stigmatised once their HCV status was known within the prison.

*“It becomes such a normal part of conversation and that really helps to destigmatise the whole thing”. (Dublin).*

*“Is there still a stigma? I ... think there is. And voicing that within the prison or in the landing can still have the impact in someone coming forward or trying to come forward. How do you keep that confidential? How do they approach someone letting them know they can come and get tested without it being broadcasted across the landing*”? (Non-Dublin).

*“You’d see that’d be the beauty in universal testing, because then it’s expected. It would destigmatise it”*. (Dublin).

### Inter-prison variations in prisoner health needs and health service delivery and priority

A consistent narrative emerging form the focus group discussion was a recognition of the heterogeneity of prison populations linked with differences in how health care was delivered at different locations. Numerous references were made regarding Dublin prisons “inside the pale” versus other prisons and how resources were allocated. The tone of the narrative revealed a subtle undertone that Dublin based prisons have more resources allocated to them compared to other prisons. Some participants regarded resource allocation as “urban centric”.

*“Not a lot of them, very little of them want screening and wouldn’t classify themselves at risk of having picked it up. One is probably the most we’d ever had on treatment”.* (Non-Dublin).

*“In my experience, very little, one in eight years since I’ve been there. We do a moderate amount of screening and it doesn’t turn up”.* (Non-Dublin).

*“I feel that prisoners are not getting treatment because they’re outside of the Dublin area*”. (Non-Dublin).

“Feel that they are prejudiced because they are outside of the M50”. (Dublin).

*“It is very urban centric and then you get out of the urban areas … more rural… and we are not as invested and it is not as high profile”.* (Non- Dublin).

### Linkage to care

A number of participants reported challenges with linkage to care and the short prison sentences served. This was of particularly challenging in remand and female prisoners.

*“We screen, screen, screen. You know it’s almost like a routine but its moving it on to treatment is the problem”.* (Dublin).

*“We’re doing a lot of screening which throws up a lot of people … but where to next?”* (Dublin).

*“They ‘re hardly in but they are gone home .... what do we do with the positive test then”. (*Non-Dublin).

*“We have a very nomadic type of client group so to try and have them for the required time that is required to finish treatment you know... huge problems with a full course of treatment ...50% remand”*. (Dublin).

Participants who had access to in-reach hepatology services found that this had a positive impact on linkage to care. The presence of in-reach hepatology as seen as having the added benefit of increasing awareness of HCV a and keeping it a priority in prisons where it was available. This service was only available in three of the fifteen locations included in the study. The use of tele -medicine was seen as a facilitator to treatment uptake. It allowed easy and timely access to consultant care and reduced the need for patients to be brought to hospital reducing cost, risk and staffing requirements.

*“Doing it (treatment*) *in house makes a big difference because we have a hard to reach group, a high-risk cohort”.* (Dublin).

*“We have developed that a little bit recently with a telemedicine link so we’ve reduced our transport needs for consultant reviews*”. (Dublin).

*In reach has transformed that. It’s hugely important, that accessibility. That changes how people think. It’s foremost in people’s minds. They’re being seen. That makes a huge difference*. (Dublin).

*“The other thing we should be looking at is shared learning. Something like the echo model is something that would really be valuable in all prisons”.* (Dublin).

### Timing of screening

There were mixed views on the timing of screening. A number of participants favoured a routine, opt-out approach at committal others expressed concerns about prisoners having competing priorities at this time. Concerns were also expressed regarding staffing levels, time allocated to the committal process and the presence of security staff compromising confidentiality.

*“One of the things to get rid of the barriers is making it routine at committal, you wouldn’t think twice. That’s probably where we need to get to. The same way as you expect to give urine”.* (Non-Dublin).

*“We don’t have protected time for committal interview and that is a significant barrier to changing any type of comprehensive screening. The opportunity is lost. And you also have an officer standing in the room so... ”.* (Dublin).

*“They’re all actively seeking medication...screening blood tests is so far down the line of what they are thinking. Day after still all over the place... two weeks later better when they ‘re settled...”.* (Dublin).

### Stability of prison life

All participants reported stability of prison life and access to medical care as a facilitator to prisoners’ engagement in HCV care. Barriers to community health care access and the chaotic pre-incarceration lifestyles of many prisoners could be successful addressed and overcome in prison.

*“Accessibility and stability. Access to nursing is 24/7, looking at holistic ... monitor weight loss ... all these things that have come up maybe in the community ... not the easiest to engage in health care”.* (Non-Dublin).

*“So, the stability ... the chaotic lives stop and the support network ... can wrap around that individual”.* (Dublin).

## Discussion

This study provides a unique insight into Irish nurse managers’ views on HCV screening and treatment in Irish prisons and other closed settings. Many of the themes identified in this research have been reported previously in the literature with regard to clinical provision and prison staff. Lack of knowledge, fear of treatment and concerns regarding stigma are known barriers to HCV infected PWID and prisoners engaging with HCV screening and treatment [31–33, 42]. Many of these barriers are historical, related to interferon based treatments and have been removed by the newer DAA therapies [43–45]. Prison nurses are uniquely placed to engage prisoners in educational programs and health promotion is viewed as a core component of prison health care and prison nurse duties [20, 40]. Identified opportunities for health promotion in prisons include, advice on prevention of communicable diseases, modifying high-risk behaviours and measures to improve mental health [20]. Making HCV information leaflets available in all relevant languages across the IPS has the potential to increase awareness and engagement in HCV care among Irish prisoners.

Prisons are designed for punishment, correction, rehabilitation and return to the community which at times can impact on the goals of health care. The concept of dual loyalty has been described in the literature and can cause conflict between security and health care staff [46]. This study reports the challenges that security requirements can pose on prisoner access. This was of particular relevance for protection prisoners, a growing cohort in Irish prisons due to an increasing gangland culture in Ireland [47]. Clinical independence is an essential component of health care provision and professionalism. The relationship between prison health care provider and prisoner is unequal and not based on free choice. The aims of incarceration can, at times, be in conflict with the provision of optimal prison healthcare. Independence of health care provision is recognised as a critical element for quality health care in prisons and is underpinned by international standards [20, 46, 48]. Many prisons fail to meet these standards because of, a lack of awareness, legal regulations, contradictory terms of employment for health care providers, or poor health care governance structures [48]. Irish nurses did not feel compromised in their clinical work and describe a respectful and collaborative relationship with their security colleagues. Irish nurse managers felt supported by prison officers and governors and describe a whole prison approach to health care delivery in Irish prison as recommended by the WHO [20].

This study reports on the inter-prison variation in prisoners’ health needs, levels of communicable disease, health care priorities and health care delivery in Irish prisons. Prisoners are often reported in the literature as a single homogenous group but it is recognised that there is much heterogeneity in prison populations globally [14, 19, 49]. This variation can be regional, between countries and even within different prisons in the same country. There is much variation in HCV infection in prisons globally and is directly linked with the numbers of PWID incarcerated at the location [16, 17]. This variation was reported in the two previous Irish prison HCV studies and prison in Ireland are categorised in low, medium and high-risk prisons [26, 50]. The majority of high risk prisons are Dublin based [26, 50]. This variation in HCV prevalence and numbers of prisoners with a history of IDU within Irish prison supports an institution specific response to screening and treatment informed by local community -based HCV treatment services, available resources and existing structures of health care delivery.

This study highlights different approaches to and levels of HCV screening in Irish prisons. These variations are dependent on the perceived levels of HCV infection, available staffing and structures of health care delivery at different prison locations. The focus group narrative suggests an ad-hoc approach to screening, alongside difficulties in providing the treatment care continuum. Recent National HCV Screening Guidelines recommend that all prisoners should be offered HCV screening on entry to prison [28]. There was limited support within the focus group for this approach. A small number supported an opt-out approach to HCV screening at committal but the majority of participants expressed concerns about its feasibility. Staff shortage, limited time dedicated to the committal process, prisoners competing priorities at committal and concerns regarding confidentiality were identified as barriers. Fear, poor patient venous access, lack of training and confidence in phlebotomy skills and concerns for personal safety were other barriers identified to HCV screening by nurse managers.

It is recognised that venipuncture can be challenging in PWID due to poor venous access linked to intravenous drug use and associated medical complications [51]. Poor vascular health may require specialist staff to take blood, which if only available in hospital phlebotomy services can increase stigma, cost and security concerns for prisoners [52]. This identified barrier can be removed by the use of dried blood spot (DBS) testing which is non-invasive and can be performed by clinical and non-clinical staff [53]. Two UK studies showed that offering DBS testing within specialist addiction services and prisons led to a threefold to six-fold increase in HCV testing [53, 54]. A recent systematic review identified DBS as the best available targeted intervention for increasing HCV case-finding among PWID [55]. This approach to screening is cost -effective in prisons if continuity of treatment/care is ensured [56]. DBS testing should be considered in Irish prisons and has the potential to remove many of barriers to HCV screening identified in this study. Prisoners concerns regarding stigma was identified as a barrier to HCV care by many participants. Concerns regarding stigma experienced by HCV infected PWID and prisoners are well documented [31, 35, 42, 57]. Maintaining complete medical confidentiality in prison settings can be a challenge [20]. Disclosure of a prisoner’s HCV status may occur due to their attendance at certain clinics or having certain blood tests performed. Adopting a standardised opt-out approach to HCV screening at committal across the IPS has the potential to increase screening uptake, reduce the stigma associated with declaring IDU and increase confidentiality [11, 58].

This study also highlighted the extra challenges encountered with remand prisoners. Like most prisoners globally the majority of Irish prisoners serve short prison sentences [7, 25, 59, 60]. This is even more problematic among remand prisoners. Historically HCV treatment lengths were greater than sentence length. The advent of short acting pangenotypic DAA regimes has now revolutionised HCV treatment within prison settings [61, 62]. Reducing treatment times to 8 weeks without negatively impacting treatment outcomes allows many more prisoners to complete treatment before release. Short prison sentences, while challenging, could also be viewed as an opportunity since large numbers of at risk and HCV infected people come in contact with the criminal justice system annually, providing a unique public health opportunity to engage this underserved and hard to reach cohort.

This study identifies linkage to care as a challenge and reports a number of facilitators that improved treatment uptake post screening. These include in-reach hepatology services and the use of tele-medicine. These have been previously identified at enabling and improving linkage to care [63, 64]. A comprehensive approach to prison HCV care has the potential to impact positively on community HCV management, but its effectiveness is dependent on community linkage on release [65] Transitioning from prison to community is seen as a high-risk time for prisoners as they adapt to their community on release [7, 66]. Ensuring that prisoners who have been screened or started treatment for HCV are linked to community services underpins the cost-effectiveness of most screening and treatment models adopted for prison HCV management [56, 58, 67].

This study highlights the complexity of peer involvement in prison health care. Previous studies have shown that the use of peer workers in community-based HCV treatment has a positive impact on the uptake of services [8, 68]. Research shows high levels of satisfaction among service users and staff in community-based drug treatment clinics with this role [68, 69]. Peer workers can dispel the myths and fears associated with HCV treatment, reduce stigma, enhance mutual trust, increase social support, and increase knowledge and engagement in HCV care [34, 68, 70].

A large 2015 systematic review (mainly qualitative studies) of peer education and support in prison settings found that peer education interventions are, effective at reducing risk behaviours, acceptable within the prison environment and have a positive impact on prisoner wellbeing [69]. Peer workers have, the ability to connect with other prisoners, reduce social stigma and impact positively with a vulnerable patient cohort who are traditionally resistant to professional advice [69, 71, 72]. There are also direct benefits for the peer workers themselves and benefits for the wider prison system including, more effective use of resources and the ability to expand the range of prison-based health services available to inmates [72]. Research into cost-effectiveness is sparse [69]. Peer interventions in prisons can impact positively on health outcomes, but these effects are more well-defined for peer deliverers [69, 71, 73]. There is evidence to suggest that prison peer workers can be subjected to “burnout” and that supervisory processes need to be considered carefully in order to avoid the intervention from being counter-productive [73]. It is recognised that peer interventions can have adverse effects on the security of prisons [71, 72]. This security risk requires organisational support within the prison to ensure smooth implementation and safety of prisoners. Peer interventions need to be incorporated into prison life and require a collaborative approach between prison health care providers and prison services to be delivered effectively [72].

The health needs of prisoners are diverse and can include: addiction, mental health and management of communicable diseases [18, 23, 33]. Prison healthcare provision is challenging for all staff and particularly for nurses who have to adapt to the security requirements of prison life. Prison healthcare is an essential aspect of incarceration. Healthcare (and nursing) in prisons in Ireland is based on a clearly-defined legal framework (the ‘Prison Rules’) and the IPS has statutory responsibility for its delivery [74]. IPS nurses have a broad range of professional experience and qualifications. Their clinical work is diverse and includes multiple areas of responsibility. The unique skill set of Irish prison nurses was identified in this study. Participants reported that Irish prison nurses were uniquely placed to manage and care for prisoners in a holistic fashion and with their knowledge and understanding of local prison regimes were well placed to upscale HCV care across the Irish prison estate. This study highlighted a deficit in phlebotomy training among some nurses. This is of concern given the high rates of BBV infections and the risk of transmission through needle stick injury [16, 75]. This training deficit will need to be addressed by the IPS as a matter of urgency. Participants did not address knowledge or experience of post exposure prophylaxis ((PEP) in the focus group. This is an area that warrants further investigation.

The use of focus group methodology allowed this study to have a national coverage which is a major strength with regard to the generalisablity of its findings. It is also a practical and acceptable way to conduct research in real life prison settings. The interaction among participants during the group provided the opportunity for an in-depth exploration of topics.

Researcher 1 (DC) was known to most focus group participants which may have impacted on their willingness to fully disclose their views. The recruitment of only nurse managers as participants may not reflect the experiences of front-line prison nurses so its findings may not fully represent the views of Irish prison nurses on HCV in Irish prison. The researchers reported the findings in a manor to ensure the confidentiality of all participants so reporting the narratives as either Dublin or Non -Dublin does not fully reveal the uniqueness and differences between different prison settings. Demographics such as age, gender and length of time in service were not collected since these were deemed to be too sensitive to collect in a work environment and so we cannot report and compare the narratives according to age, gender or of length of time in service.

## Conclusion

Nurses are the main providers of health care in Irish prisons. They work in a very complex work environment where they have to navigate the struggle between custody and caring. Often correctional priorities override nursing and health care priorities. Uniquely they face safety and security concerns daily. This study identifies many challenges to prison-based HCV screening and treatment including; fear of treatment and stigma, lack of knowledge, reduced staffing, security and custodial requirements taking priority over health care delivery, poor venous access coupled with poor phlebotomy skills, short sentence length and linkage to care. Identification of these barriers can inform changes to Irish prison health care practice and policy including; the introduction of DBS, opt-out screening at committal, the use of 8-week pangenotypic DAA regimes as close to committal as possible and the expansion of in-reach hepatology services and tele-medicine. The recognised heterogeneity of prison populations and associated differences in health care delivery will require a location specific approach to HCV management. Prison peer workers have the potential to impact positively on the delivery of health and HCV care in prisons but needs a well organised structure to provide support, governance and manage security concerns. Engaging nurse managers and nurses in the planning and implementation of prison-based HCV management will optimise the public health opportunity that incarceration provides.

## Abbreviations

BBV: Blood-borne virus
DAA: Direct-acting antiviral
DBS: Dried blood spot
HCV: Hepatitis C Virus
IDU: Injecting drug use
IPS: Irish Prison Service
MSM: Men who have sex with men
PEP: Post exposure prophylaxis
PIL: Participant information leaflet
PWID: People Who Inject Drugs
WHO: World Health Organisation
